# *Haemophilus influenzae* type b as an important cause of culture-positive acute otitis media in young children in Thailand: a tympanocentesis-based, multi-center, cross-sectional study

**DOI:** 10.1186/1471-2431-14-157

**Published:** 2014-06-20

**Authors:** Pavinee Intakorn, Nuntigar Sonsuwan, Suwiwan Noknu, Greetha Moungthong, Jean-Yves Pirçon, Yanfang Liu, Melissa K Van Dyke, William P Hausdorff

**Affiliations:** 1 Department of Otolaryngology, Queen Sirikit National Institute of Child Health, 420/8 Rajvithi Road, Rajthevee, Bangkok 10400, Thailand; 2 Department of Otolaryngology, Faculty of Medicine, Chiang Mai University, 110 Intawaroros Road, Muang District, Chiang Mai 50200, Thailand; 3 Department of Otolaryngology, Hatyai Hospital, 182 Ratakan Haiyai, Songkhla, 90110, Thailand; 4 Department of Otolaryngology, Phramongkutklao Hospital of the Royal Thai Army, 315 Rajvithi Road, Rajthevee, Bangkok, Thailand; 5 GlaxoSmithKline Vaccines, Avenue Fleming 20, 1300 Wavre, Belgium; 6 GlaxoSmithKline Vaccine Singapore, 150 Beach Road, Gateway West, 22-00, 189720 Singapore, Singapore; 7 Current affiliation: Janssen Pharmaceutical companies of Johnson and Johnson, 2 International Business Par, 07-00, The Strategy, Singapore 609930, Singapore; 8 Current affiliation: Amgen, Inc., 1 Amgen Center Dr, Thousand Oaks, CA 91320, USA

**Keywords:** Acute otitis media, Hib, *Streptococcus pneumoniae*, *Haemophilus influenzae* and antibiotic resistance

## Abstract

**Background:**

*Streptococcus pneumoniae* (*S. pneumoniae*) and *Haemophilus influenzae* (*H. influenzae*) are considered major causes of bacterial acute otitis media (AOM) worldwide, but data from Asia on primary causes of AOM are limited. This tympanocentesis-based, multi-center, cross-sectional study assessed bacterial etiology and antimicrobial susceptibility of AOM in Thailand.

**Methods:**

Children 3 to 59 months presenting with AOM (< 72 hours of onset) who had not received prescribed antibiotics, or subjects who received prescribed antibiotics but remained symptomatic after 48–72 hours (treatment failures), were eligible. Study visits were conducted from April 2008 to August 2009. Bacteria were identified from middle ear fluid collected by tympanocentesis or spontaneous otorrhea swab sampling (< 20% of cases). *S. pneumoniae* and *H. influenzae* serotypes were determined and antimicrobial resistance was also assessed.

**Results:**

Of the 123 enrolled children, 112 were included in analysis and 48% of the 118 samples were positive for *S. pneumoniae (23%* (27/118))*, H. influenzae (18%* (21/118))*, Moraxella catarrhalis (6% (7/118)) o*r *Streptococcus pyogenes (3% (4/118)).* The most common *pneumococcal* serotypes were 19F (26%) and 14 (22%). The majority of *H. influenzae* isolates were encapsulated (18/21), with 13 type b (Hib) representing 62% of all *H. influenzae* isolate or 11% of all samples (13/118), and there were only 3 non-typeable isolates. Despite high antibiotic resistance, amoxicillin/clavulanate susceptibility was high. No pneumococcal vaccine use was reported.

**Conclusions:**

*S. pneumoniae* and *H. influenzae,* both frequently antibiotic resistant, were leading causes of bacterial AOM and there was an unexpectedly high burden of Hib in this population unvaccinated by any Hib conjugate vaccine. Conjugate vaccines effective against pneumococcus and *H. influenzae* could potentially reduce the burden of AOM in this population.

## Background

Acute otitis media (AOM) is one of the most frequent bacterial infections in children, and one of the primary reasons for the prescription of antibiotics by pediatricians [1,2]. *Streptococcus pneumoniae* (*S. pneumoniae*) and non-typeable *Haemophilus influenzae* (*H. influenzae)* have historically been considered the leading causes of bacterial AOM [3]. Following introduction of the 7-valent pneumococcal conjugate vaccine (PCV7), in the United States, a relative increase in non-PCV7 serotypes and non-typeable *H. influenzae* (NTHi) was observed. There were few cases of AOM due to *Moraxella catarrhalis* (*M. catarrhalis*) or *Streptococcus pyogenes* (*S. pyogenes*) and no reported cases due to *H. influenzae* type b (Hib) [4]. Even prior to the Hib vaccination era, encapsulated *H. influenzae* was rarely reported as a cause of AOM in the United States [3].

Most data on the topic come from North America and Europe, however, and studies of the burden, etiology and societal impact of AOM in Asia are sparse. While some studies suggest a low estimated prevalence [5,6] and a lower physician-reported frequency of AOM visits in Asia than elsewhere [7], others have highlighted the importance of AOM in the region [6]. The significant regional burden of chronic suppurative otitis media [8], a complication of AOM, suggests that AOM is indeed of public health concern.

Regional treatment patterns of AOM may also raise concerns given the extremely high rates of penicillin non-susceptibility of *S. pneumoniae* isolates and of ampicillin/amoxicillin resistance for *H. influenzae* non-invasive isolates documented in young children in East Asia [9-11]. A recent survey reported that most of the physicians in Asian countries use oral antibiotics as part of first line treatment of AOM [7], despite ‘watchful waiting’ recommendations in many countries across the world [12,13].

There is thus a need for AOM etiology data in the region, ideally from tympanocentesis samples, as data extrapolated from pathogen distribution from nasopharyngeal samples do not necessarily represent pathogen distribution in the middle ear [14,15]. This study aimed to add to the limited AOM data in Thailand, to characterize the bacterial etiology and serotypes of AOM cases in young children in Thailand, where both Hib and pneumococcal conjugate vaccine use are reported to be only <5% [16,17], and to determine antibiotic susceptibility of the pathogens. These data could have important clinical implications for determining the best approach for prevention and treatment of AOM in Thailand [18,19].

## Methods

### Study design

This was a tympanocentesis-based, multi-center, cross-sectional study conducted within a routine clinical setting in several regions of Thailand: 2 centers in Bangkok, one in Hatyai in southern Thailand and one in Chiang Mai in northern Thailand. Target enrollment was at least 100 patients over a year, based on the assumption that in the context of high antibiotic use, 40% of samples would be culture positive [3,4,20]. The study included children 3 to 59 months of age visiting Ear Nose and Throat (ENT) clinics for AOM, and from whom a middle ear fluid (MEF) sample was available either by tympanocentesis or careful sampling of spontaneous otorrhea which occurred less than 24 hours prior to the visit. Eligible patients were either subjects with a new episode of AOM (less than 72 hours since onset of symptoms) who had not yet received any antibiotics prescribed by a physician, or subjects who were diagnosed with AOM within 48–72 hours prior to study enrollment, received antibiotic therapy from a physician, but remained symptomatic at the time of study entry (treatment failures). Patients who received systemic antibiotic treatment for a disease other than AOM in the 72 hours prior to enrollment, and patients receiving antimicrobial prophylaxis for recurrent AOM, defined as at least 3 episodes in the past 6 months or 4 episodes in the past 12 months, were excluded. Children who were hospitalized during the diagnosis or treatment of AOM were also excluded. All study visits took place between 2 April 2008 and 28 August 2009.

During screening and enrollment, ENTs maintained a logbook to collect anonymized demographic information for subjects 3 to 59 months of age who were diagnosed with AOM to determine the representativeness of the AOM patients who were included in the study. ENTs obtained informed consent from parents/guardians of eligible children prior to performance of any study-specific procedures. Once enrolled, demographics, medical history, care history and general symptoms were collected and a clinical examination was performed; AOM was diagnosed after otoscopic examination of the tympanic membrane by the ENT and was classified according to the otoscopy score (8 grades) (OS-8), which measures the severity of tympanic-membrane inflammation. The OS-8 scale is only appropriate for use in children with an intact tympanic membrane, and therefore was not used for children with otorrhea. Spontaneous otorrhea or an OS-8 score of at least 2 was necessary for the child to meet AOM diagnosis criteria. The levels of the OS-8 scale from level 2 are as follows: 2 indicates hyperemia, air-fluid level, no opacification, meniscus noted; 3 indicates hyperemia, complete effusion, no opacification; 4 indicates hyperemia, opacification, air-fluid level observed, no bulging; 5 indicates hyperemia, complete effusion, opacification, and no bulging; 6 indicates hyperemia, bulging rounded doughnut appearance of tympanic membrane; 7 indicates hyperemia with bulla formation.

### Middle ear fluid sample collection and sample analysis

MEF samples were collected by performing tympanocentesis. In cases of otorrhea, investigators were advised to remove and clean the ear canal material, and deep aspiration of the MEF material, via needle insertion, was attempted to avoid contamination and spurious results. Since pathogen distribution from tympanocentesis and otorrhea may differ [21], the study protocol limited otorrhea samples to represent no more than 20% of all subjects.

Samples were kept in Amies transport media and transferred to the central laboratory within 16 hours for plating at room temperature. Analysis of samples was performed at a central laboratory to isolate bacterial pathogens, assess serotypes and determine the antimicrobial susceptibility profile. MEF samples were inoculated in chocolate agar and blood agar with gentamycin and otorrhea samples were inoculated in chocolate agar with bacitracin and blood agar with gentamycin. *S. pneumoniae* serotyping was performed through polymerase chain reaction (PCR) [22] and *H. influenzae* serotyping was performed through monovalent antisera a, b, c, d, e and f at the International Emerging Infections Program of the United States Centers for Disease Control and Prevention. After initial serotyping of *H. influenzae* isolates the results were confirmed by a second laboratory which was blinded to the initial serotyping results. Definitions of antimicrobial susceptibility were based on the Clinical and Laboratory Standards Institute 2009 standards [23]. Susceptibility to the following antibiotics was assessed: penicillin, amoxicillin/clavulanate, cefuroxime, cefotaxime, erythromycin, azithromycin, ampicillin, chloramphenicol, tetracycline, levofloxacin and trimethoprim/sulfamethoxazole.

### Statistical analysis

Children with bilateral infections were considered a single episode but had 2 samples collected, one from each ear. Descriptive statistics were used to compare demographics, clinical characteristics, pathogen distribution and antibiotic susceptibility among enrolled children. All statistical analyses were performed using SAS, version 9.1 or later (SAS Institute Inc., Cary, NC, USA), and Microsoft Excel (2002 SP3 or later), for graphical purposes.

### Ethical approval

The study protocol was reviewed by the ethical review committees of all participating hospitals and the Ethical Review Committee for Research in Human Subjects at the Thailand Ministry of Public Health.

## Results

### Study subjects

**Figure 1:**
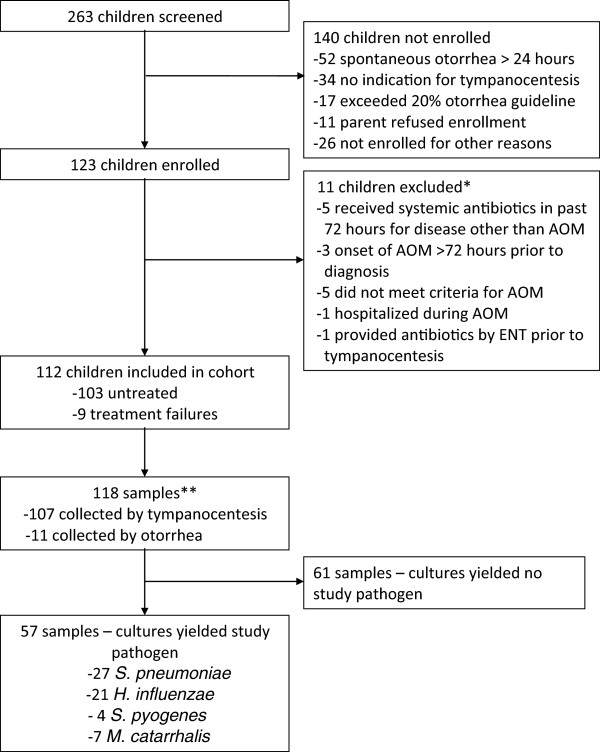
Enrollment and etiology of AOM patients included in the study.

Study visits took place for 123 children experiencing AOM among 263 screened children (Figure 1). One hundred and twelve children fulfilled study criteria. Nine of the 112 (8%) children were classified as treatment failures. Six of the 112 children were experiencing bilateral infections for which samples from both the left and right ears were collected. Of the 118 samples collected, 91% (107/118) were collected by tympanocentesis. The primary reason for non-enrollment was spontaneous otorrhea more than 24 hours prior to the visit (n = 52).

### Demographic characteristics and clinical history

The median age of screened children was 33.5 months compared to a median age of 36 months among participating children (range 5–59 months) (Table 1). Nine percent (10/112) of participating children were between 3 and 11 months of age, 14% (16/112) were between 12 and 23 months, and the remainders were uniformly distributed between the other classes of age (24–35, 36–47 and 48–59 months). Fifty-five percent (62/112) of participating children were females. Sixty-four percent (7/11) of children with spontaneous otorrhea were less than 24 months of age, while 19% (19/101) of children in whom tympanocentesis was used were less than 24 months. None of the children had received any doses of a pneumococcal conjugate vaccine, while 4% (5/112) had received at least one dose of influenza vaccine. Antibiotic use within the past month was reported for 23% (26/112) of children. AOM was classified as recurrent for 7% (8/112) of children.

**Table 1:** AOM pathogens analyzed by age group, gender, and sample collection method

	**Total (positive and negative)**	**Any culture positive**	***S. pneumoniae***	**Non-Hib *H. influenzae***	**Hib**	***M. catarrhalis***	***S. pyogenes***
**Age**							
3–11 months	10	5 (50%)	2	1	1	0	1
12–23 months	16	10 (63%)^1^	2	1	5^1^	1^1^	2
24–35 months	29	11 (38%)	5	2	2	2	0
36–47 months	28	14 (50%)	8^2^	0	4	2	0
48–59 months	29	16 (55%)^3^	9^3^	4^3^	1	2	1
**Total episodes**	112	56 (51%)^1,3^	26^2,3^ (23%)	8 (7%)^3^	13 (12%)^1^	7 (6%)^1^	4 (4%)
**Male**	51	26 (51%)	10^2^ (20%)	5 (10%)	7 (14%)	1 (2%)	3 (6%)
**Collection method**							
Otorrhea	11	6 (55%)	1	1	1	0	3
Tympanocentesis	107	51 (48%)	26^4^	7	12	7	1
**Total samples**	118^4^	57 (48%)	27 (23%)^4^	8 (7%)	13 (11%)	7 (6%)	4 (3%)

Data presented per episodes in the upper part of the table and per samples in the lower part. Percentages are calculated based on the total (positive and negative) number of episodes or samples respectively.

^1^Includes one episode with a co-infection by *H. influenzae* and *M. catarrhalis.*

^2^Includes one episode with a bilateral infection, from which the two collected samples were culture positive for *S. pneumoniae* (unknown serotype, same susceptibility to antibiotics).

^3^Includes one episode with a co-infection by *S. pneumoniae* and *H. influenzae.*

^4^Two samples were collected from the 6 children presenting with a bilateral infections, leading to a total of 118 samples from the 112 episodes. No bacteria were identified in 5 bilateral infections, the last one was positive for *S. pneumoniae* (27 samples from 26 episodes).

### Microbiology

**Figure 2:**
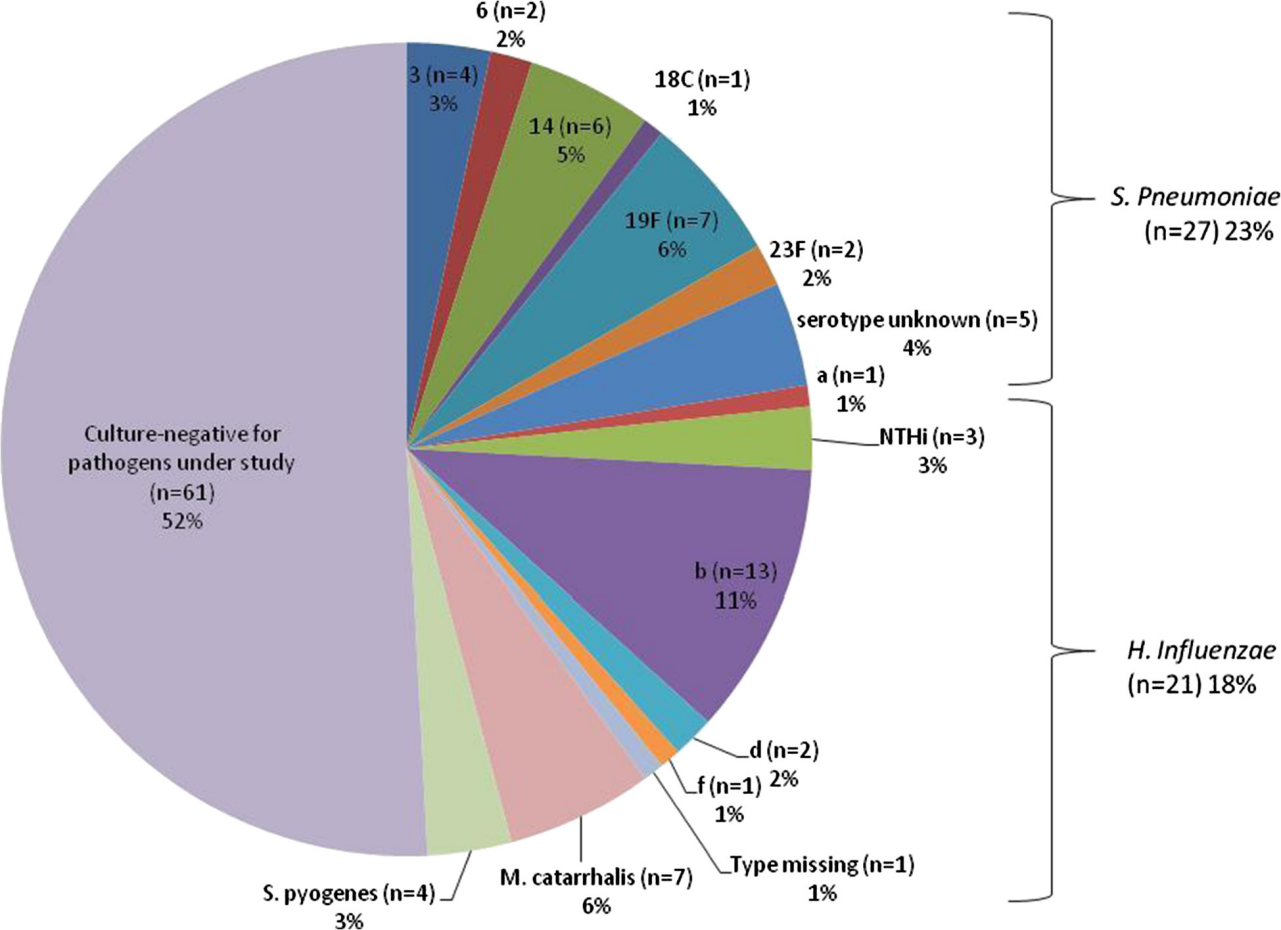
**Culture results and pathogens under study identified from middle ear fluid samples (N = 118).** Culture results from middle ear fluid samples including serotype distribution for *S. pneumoniae* (*Spn,* n = 27*)*, and *H. influenzae* (*H. inf,* n = 21*).* There were two co-infected samples due to one co-infection of *S. pneumoniae* 23F and *H. influenzae* serotype a, and one co-infection of Hib and *M. catarrhalis.*

Overall, 48% (57/118) of samples yielded cultures with one of the 4 bacterial pathogens under study (*S. pneumoniae, H. influenzae, M. catarrhalis* or *S. pyogenes)* (Figure 2), 2 of which were positive for more than one bacteria*.* The most frequently detected bacteria was *S. pneumoniae* (47% (27/57)), followed by *H. influenzae* (37% (21/57)), *M. catarrhalis* (12% (7/57)) and *S. pyogenes* (7% (4/57)). Among the 11 samples collected from otorrhea episodes, one was positive for *S. pneumoniae,* 2 for *H. influenzae* and 3 for *S. pyogenes.* Two of the 9 treatment failure samples were positive for a pathogen under study, both of which were *S. pneumoniae.* The most common pneumococcal serotypes isolated from the 27 *S. pneumoniae*-isolates were 19F (7/27 (26%)), 14 (6/27 (22%)) and 3 (4/27 (15%)) (Figure 2). Out of the 21 *H. influenzae* isolates, 13 (62%) were serotype b (Hib), 3 (14%) were non-typeable, and the remainders were serotypes a (1 isolate (5%)), d (2 isolates (10%)) and f (1 isolate (5%)), with one (5%) missing (Figure 2). Overall, Hib was detected in 11% of all samples (13/118). The 2 co-infected samples were due to one co-infection of *S. pneumoniae* 23F and *H. influenzae* serotype a, and one co-infection of Hib and *M. catarrhalis.*

*S. pneumoniae* and Hib and non-Hib *H. influenzae* were the most commonly detected pathogens in all age groups (Table 1). In the youngest age range of 3–11 months, *S. pneumoniae* and *H. influenzae* were each isolated from 2/10 episodes (20%). Among children 12–35 months of age, *S. pneumoniae* was isolated from 7/45 (15.5%) episodes while *H. influenzae was* detected in 10/45 (22%). In the oldest children, 36–59 months of age, *S. pneumoniae* was detected from 17/57 (30%) episodes, and *H. influenzae* from 9/57 (16%). Potential risk and protective factors, including premature birth, HIV infection, child care attended, child breast-fed and number of household siblings less than 5, were similar when compared by pathogen (data not shown). Due to small numbers, the differences in age group and potential risk factors by pathogen were not tested for statistical significance.

### Symptoms

The most frequently reported symptom was ear pain, reported for 95% (106/112) of episodes, followed by irritability, reported for 49% (55/112) of episodes (Table 2). Fever was reported for 12% (3/26) of children experiencing AOM due to *S. pneumoniae* but was not reported for any children experiencing AOM due to Hib or non-Hib *H. influenzae.* Trouble sleeping was reported for 2% (2/26) of children experiencing AOM due to *S. pneumoniae*, 31% (4/13) of those experiencing AOM due to Hib and 25% (2/8) of those experiencing AOM due to non-Hib *H. influenzae* (Table 2). Due to small numbers, the differences in symptoms by pathogen were not tested for statistical significance.

**Table 2:** Symptoms reported at the visit for AOM patients in the study

	***S. pneumoniae *** **positive (N = 26)**	**Non-Hib *H. influenzae *** **positive (N = 8)**	**Hib positive (N = 13)**	**Total (N = 112)**
Ear pain	25 (96%)	8 (100%)	13 (100%)	106 (95%)
OS-8 > 5	15 (58%)	4 (50%)	10 (77%)	64 (57%)
Irritability	16 (62%)	0 (0%)	7 (54%)	55 (49%)
Tugging	6 (23%)	1 (13%)	4 (31%)	33 (29%)
Temperature – axillary				
37.5-39.0°C	12 (46%)	2 (25%)	3 (23%)	30 (27%)
> 39.0°C	3 (12%)	0 (0%)	0 (0%)	6 (5%)
Trouble sleeping	2 (8%)	2 (25%)	4 (31%)	28 (25%)
Anorexia	4 (15%)	0 (0%)	1 (8%)	16 (14%)
Vomiting	3 (12%)	0 (0%)	1 (8%)	10 (9%)
Diarrhea	0 (0%)	0 (0%)	1 (8%)	4 (4%)
Hearing loss	1 (4%)	0 (0%)	2 (15%)	3 (3%)
Conjunctivitis	0 (0%)	0 (0%)	0 (0%)	2 (2%)
Lethargy	2 (8%)	0 (0%)	0 (0%)	2 (2%)

N = number of episodes, data presented as n (%).

### Hib-positive AOM

Thirty-eight percent (5/13) of Hib-positive AOM and 13% (1/8) of AOM due to other *H. influenzae* were in children 12–23 months, compared to 14% (16/112) of AOM overall. Fifteen percent (2/13) of children with Hib-positive AOM and 13% (1/8) of children with AOM due to other *H. influenzae* reported taking antibiotics in the past month. Two of the 3 children who experienced hearing loss had Hib-positive AOM. Irritability and ear tugging were reported for a greater proportion of children with Hib-positive AOM compared to children with AOM due to other *H. influenzae* (54% (7/13) and 31% (4/13) versus 0% and 13% (1/8), respectively). Seventy-seven percent (10/13) of Hib-positive and 50% (4/8) of other *H-influenzae*-positive children had an OS-8 scale score of greater than 5.

### Antibiotic susceptibility

Among the 27 *S. pneumoniae* isolates, all were susceptible to amoxicillin/clavulanate and to penicillin, 11% (3/27) were non-susceptible to cefotaxime, 63% (17/27) were non-susceptible to cefuroxime, 67% (18/27) were non-susceptible to erythromycin and 78% (21/27) were non-susceptible to trimethoprim/sulfamethoxazole (Table 3). Eighty-one percent (22/27) of *S. pneumoniae* isolates were multidrug resistant. Among 19F isolates, the most prominent serotype, 2 out of 7 were non-susceptible to cefotaxime and 5 out of 7 were non-susceptible to cefuroxime. All *H. influenzae* isolates were susceptible to amoxicillin/clavulanate and to cefotaxime, 5% (1/21) was non-susceptible to cefuroxime, and 20% (4/20) were non-susceptible to ampicillin, with ampicillin data missing for one isolate (Table 3). Three of the 4 isolates not susceptible to ampicillin were Hib isolates. One (Hib) of the 21 *H. influenzae* isolates was beta-lactamase-negative ampicillin-resistant but susceptible to amoxicillin/clavulanate.

**Table 3:** **Antibacterial non-susceptibility of *S. pneumoniae *** **and *H. influenzae *** **isolates**

	**Number of non-susceptible**^**1 **^**isolates**	
**Antibiotic**	***S. pneumoniae *** **isolates (N = 27)**^**2**^	***H. influenzae *** **isolates (N = 21)**
Amoxicillin/Clavulanate	0 (0%)	0 (0%)
Ampicillin^3^	-	4 (20%)
Azithromycin	26 (96%)	2 (10%)
Cefotaxime	3 (11%)	0 (0%)
Cefuroxime	17 (63%)	1 (5%)
Chloramphenicol	7 (26%)	2 (10%)
Erythromycin^4^	18 (67%)	-
Levofloxacin	0 (0%)	0 (0%)
Penicillin^4^	0 (0%)	-
Tetracycline	18 (67%)	2 (10%)
Trimethoprim/Sulfamethoxazole	21 (78%)	7 (33%)

^1^Intermediate or resistant based on the Clinical and Laboratory Standards Institute 2009 standards.

^2^Two isolates are coming from the same child with a bilateral infection.

^3^Ampicillin resistance data missing for one *H. influenzae* isolate. Ampicillin sensitivity was not performed for *S. pneumoniae*.

^4^For *H. influenzae*, the median value of MIC was equal to 4.0 for Erythromycin and 0.250 for Penicillin.

## Discussion

The AOM episodes seen in this study among children who sought care from ENTs in Thailand were generally non-recurrent episodes assessed by tympanocentesis. In this study environment, where there was minimal use of either Hib or pneumococcal vaccine, bacterial pathogens were an important cause of AOM. The leading causes of bacterial AOM were *S. pneumoniae* and *H. influenzae*, representing 47% (27/57) and 37% (21/57) of culture-positive samples, respectively. The majority of *H. influenzae* was serotype b (62% (13/21)). Forty-eight percent of samples were culture-positive for one of the pathogens under study, slightly lower than the 53-58% reported in other settings [24], but consistent with the assumption that isolation of bacteria may be lower in an environment with high antibiotic use [25]. Other studies have found that PCR can detect bacteria in culture-negative MEF [26], so it is possible that these pathogens play a greater role in AOM than what was detected here.

We found slightly more *S. pneumoniae* than *H. influenzae*, consistent with what was seen elsewhere in the pre-PCV7 era [3]. In this population, AOM episodes were generally comprised of relatively mild, sporadic cases, rather than severe or recurrent. *H. influenzae* was slightly more prominent than *S. pneumoniae* in children 12–23 months of age while the reverse was true in children 24–59 months of age*.* Overall, the symptom profiles and potential risk factor profiles of *S. pneumoniae* and *H. influenzae* were generally similar.

One unexpected finding in the studywas the higher than expected presence of Hib. This was a surprise in part because available data suggest a low incidence of Hib-associated invasive disease in Thailand [27], although there are concerns that existing data from Asia underestimate the true burden [28,29]. Additionally, on a global level, Hib is generally perceived not to be an important AOM pathogen. Before the introduction of the Hib vaccine in the United States, for example, Hib only represented 10% of *H. influenzae* AOM cases [30], while in our study, Hib was seen in 62% of the *H. influenzae* isolates. Another exception to the general observation that encapsulated *H. influenzae* are not important causes of AOM comes from a recent, tympanocentesis-based study in Venezuela where 31% of *H. influenzae* AOM were encapsulated a, c, d and f strains (Venezuela has universal Hib immunization) [31]. Interestingly, based on the OS-8 scale, the Thai Hib cases seemed to be slightly more severe than *S. pneumoniae* or non-Hib *H. influenzae* cases.

A second surprising finding was that the median age of children in the study was 36 months, which is unusual given that AOM incidence elsewhere generally peaks at 6–18 months of age. Since the age of the screened cohort was only slightly younger than the enrolled cohort it does not appear that there was significant bias in the final study sample (i.e., those who received tympanocentesis) compared to all children who came to the ENT with suspected AOM. While it is possible that the true burden of AOM in Thailand tends to be in older children, it also may be that younger children with AOM are more often treated at home or by general practitioners and do not tend to visit the ENT. We note that a number of children could not be enrolled because of otorrhea for greater than 24 hours, which may suggest more severe AOM or may suggest that access to prompt care is limited, by distance or other factors.

The distribution of *S. pneumoniae* serotypes was similar to what has been reported in the literature [10,32] prior to PCV introduction. The generally mild profile of AOM experienced by the children in our study may explain the slightly higher than expected proportion of *M. catarrhalis* isolates, as this pathogen is often associated with milder disease [33].

Due to the risk of treatment failures, up-to-date information on antibiotic resistance has important clinical implications for determining the best approach for treatment of AOM [19]. Our results show high levels of resistance of *S. pneumoniae* to some antibiotics commonly given in Thailand for respiratory infections (Azithromycin, Cefuroxime, Erythromycin, Tetracycline, Trimethoprim/Sulfamethoxazole), and a high level of multidrug resistance. This was consistent with results from another study in Asian countries [34], which also noted a high level of resistance to macrolides. In our study only a low rate of cefotaxime non-susceptibility was seen, likely due to the fact that cephalosporins are generally only prescribed for children presenting with severe illness (moderate to severe otalgia or fever of 39°C ) at first visit or for patients who do not respond to initial treatment. Antibiotic resistance was less common for *H. influenzae,* and was similar to previously published estimates, though our isolates had lower levels of resistance to chloramphenicol (10% versus 25%) and ampicillin (15% versus 48%) [35]. It is possible that more severe AOM cases than were seen in this study would be enriched for more resistant AOM.

Currently Hib vaccine use in Thailand is extremely limited as it is not on the Expanded Program of Immunization for Thailand [35]. Uptake of PCV7 in Thailand, which is mainly used in private settings, has also been low [10], and there were no reports of pneumococcal vaccine use in the children in our study. Two other pneumococcal vaccines, Prevenar/Prevnar 13™ (Wyeth, LLC) (PCV13) and Synflorix™ (GlaxoSmithKline Vaccines) (PHiD-CV), have been licensed in recent years, and differ from PCV7 in the inclusion of 6 (1, 3, 5, 6A, 7F, 19A) and 3 (1, 5, 7F) additional serotypes, respectively. PHiD-CV also utilizes as the predominant carrier protein an outer membrane protein (protein D) derived from *H. influenzae,* as a protein D-containing 11-valent precursor formulation of PHiD-CV was previously shown to be efficacious against both pneumococcal and *H. influenzae* AOM [36]. Efficacy of PHiD-CV itself against AOM was also recently demonstrated in another double-blind randomized clinical study [37]. Although PCV13 efficacy against AOM has not yet been assessed, such data do exist for its predecessor formulation PCV7 [38]. Of the 22 pneumococcal isolates whose serotype could be identified, at least 16 (73%) represent a serotype contained in each of the two higher valent vaccines. Serotype 3, contained only in PCV13, was also identified in 4/22 (18%) of those pneumococcal isolates, but it remains unclear whether serotype 3 disease is vaccine-preventable [39]. Our results thus suggest that either vaccine would likely prevent a significant proportion of AOM cases.

The study was successful in adding to the limited data on AOM in Thailand, but there are important limitations, including few cases in the youngest children, small sample size and lack of a clear population denominator. The study did cover several, but not all, regions of Thailand, and therefore is somewhat limited in geographical representativeness. As the use of a Hib vaccine is known to be very limited in Thailand, we did not collect individual Hib vaccination status, though it could have provided further insight into the previously unrecognized burden of Hib in AOM cases that was identified in this study. An additional limitation is that the over-the-counter availability of antibiotics in Thailand could mean that some children may have received antibiotics before the study visit, This could have decreased the proportion of culture positives, and meant that bacteria that were isolated from such patients may have been those with greater non-susceptibility. However, as it was impossible to know whether any antibiotics received in this manner were appropriate for AOM and/or provided in sufficient dosage, only patients receiving antibiotics prescribed by a physician 48–72 hours prior to the study visit were considered treatment failures, as per protocol.

## Conclusions

In summary, this assessment of AOM etiology in Thai children 3 to 59 months of age visiting ENT clinics for AOM showed an unexpectedly high burden of Hib. *S. pneumoniae* and *H. influenzae* were the leading causes of AOM across all age groups, similar to what has been seen in Europe, the United States, and Latin America, and with pneumococcal serotypes similar to those found elsewhere [32,40]. These findings contribute to the scarce tympanocentesis literature in this region, and suggest that conjugate vaccines effective against pneumococcus and *H. influenzae*, both encapsulated (Hib) and unencapsulated, may be important in attempts to reduce bacterial AOM in the region.

### Trademark

Prevnar and Prevnar 13 are trademarks of Wyeth LLC.Synflorix is a trademark of the GlaxoSmithKline group of companies.

## Competing interest

GlaxoSmithKline Biologicals SA funded all costs associated with the study and with the development and publishing of the present manuscript. GM and SN declare no conflicts of interest. PI received an institutional grant and a travel grant from the GlaxoSmithKline group of companies. NS has received a grant, travel grant funding and payment for lectures from the GlaxoSmithKline group of companies. JYP and WPH are employees of the GlaxoSmithKline group of companies. WPH own stock in GlaxoSmithKline Biologicals and is co-holder of the patent for Prevnar 13™. YFL and MVD were previously employed by the GlaxoSmithKline group of companies and had stock options.

## Authors’ contributions

PI, YL and WPH participated in the conception and design of the study and together with NS, MKV, SN and GM contributed to the development of the protocol. PI, NS, SN and GM contributed to the acquisition of data. JYP (study and project statistician), PI, GM, NS, SN, YL and MKV contributed to data processing, to the statistical analysis and to the study report. MKV contributed to the interpretation of the statistical analysis and together with NS, WPH and YL to the development of the manuscript. All authors had full access to the data, read and reviewed drafts of the manuscripts and approved its final content.

## Pre-publication history

The pre-publication history for this paper can be accessed here:

http://www.biomedcentral.com/1471-2431/14/157/prepub
